# Effects of β-adrenergic receptor antagonists on oxidative stress in purified rat retinal ganglion cells

**Published:** 2007-06-11

**Authors:** Zi-Kui Yu, Yi-Ning Chen, Makoto Aihara, Wei Mao, Saiko Uchida, Makoto Araie

**Affiliations:** 1 Department of Ophthalmology, University of Tokyo, Japan; 2 Department of Ophthalmology, Renji Hospital, Shanghai Jiaotong University School of Medicine, Shanghai, China

## Abstract

**Purpose:**

To investigate the effect of β-adrenergic receptor antagonists against oxidative stress on purified rat retinal ganglion cells (RGCs), timolol, betaxolol, carteolol and nipradilol were included in the present study.

**Methods:**

RGCs were purified using a 2 step panning procedure from postnatal days 6-8 using Wistar rats. After 72 h in culture under normal condition, RGCs were exposed to oxidative stress induced by B27 medium without anti-oxidant. To verify whether this stress is apoptotic or necrotic, Annexin V and propidium iodide were used to detect apoptotic and necrotic cells after 2 h stress. The presence of a proinhibitor for intracellular cathepsin B, and an inhibitor for thiol protease (cathepsin B/H/L, calpain), was also assessed to verify necrotic cell death event in oxidative conditions. Next, RGC cultures under oxidative stress were incubated with timolol, betaxolol, carteolol, and nipradilol added, respectively, for 24 h culture. The RGC viability in each condition normalized to that under normal condition was evaluated as live cell percentage based on total experiments of 8-15.

**Results:**

Two h after oxidative stress, Annexin V and propidium iodide positive cells increased. Increased cell death under oxidative stress was significantly reduced by inhibitors for cathepsin or calpain. These data suggest that increased cell death under the current oxidative stress was due to necrosis. Under oxidative stress for 24 h, RGC viability reduced to 52.5-60.2% as compared with normal. With 10 nM and 100 nM timolol, live cell significantly increased to 69.3% and 75.5%, respectively. Both betaxolol and nipradilol enhanced live RGCs significantly in concentration of 100 nM and 1 μM, with viability of 70.5%, 71.6%, and 70.4%, 74.7%, respectively. While with 10 nM, 100 nM and 1 μM addition of carteolol, there was no significant increase in live RGC percentage which ranged from 53.1-55.0%.

**Conclusions:**

Timolol, betaxolol and nipradilol, but not carteolol, showed neuroprotective effects against oxidative stress induced by B27 without antioxidant on purified rat RGCs at concentrations of 10 nM or higher. Although the neuroprotective mechanism of β-blockers for oxidative stress is still unknown, this additive effect may deserve future studies.

## Introduction

Oxidative stress can be viewed as an imbalance between the production and clearance of reactive oxygen species (ROS) [1]. Although the mechanism that produces ROS may differ in different conditions, an influx of Ca^2+^ is probably linked with cell damage during oxidative stress [2,3]. Retina and retinal neurons, with their relatively high oxygen consumption and constant exposure to light, are prone to oxidative stress [4,5]. Oxidative stress also may be related to the pathogenesis of glaucomatous optic neuropathy (GON) [1,6]. Thus, oxidative stress is an important factor that is studied both clinically and in the laboratory and can be correlated with both retinal disease and GON. In vivo and in vitro studies demonstrated that oxidative stress-induced retinal ganglion cell (RGC) death could be alleviated by down-regulation of the downstream signaling protein, apoptosis signal-regulating kinase 1, or by addition of anti-oxidants, such as flavonoids or cannabinoids [7-9].

β-adrenergic antagonists (β-blockers) have been widely used as intra-ocular pressure (IOP)-lowering agents for the treatment of glaucoma,, and there are many reports in the literature about their in vitro neuroprotective effects. For example, timolol, a non-selective β-blocker, reportedly alleviated retinal neuronal damage induced by ischemia in animal models [10]. In addition, timolol protected RGCs against damage induced by anoxia in mixed retinal cell cultures [11], and from damage caused by glutamate in purified cultured RGCs [12]. Betaxolol, a selective β-blocker, was reported to show protective effects on retinal cells including RGCs from ischemic and N-methyl-D-aspartate (NMDA)-induced insults in animal models [10,13], and protect retinal neurons from a glutamate insult in mixed retinal cell cultures [14]. Carteolol, a non-selective β-blocker, inhibited Ca^2+^ influx in neuronal cells at high concentrations [15,16]. Furthermore, it showed a cytoprotective effect on UV-induced corneal epithelial cell death [17]. Nipradilol, a non-selective β- and selective β1-blocker with nitric oxide (NO) releasing activity [18], has been reported to protect the retina from NMDA-induced or ischemia-reperfusion conditioned insult in animal models [19,20]. It also enhanced viability of cells in purified RGC cultures [21]. The effects of these β-blockers on oxidative stress-induced RGC damage, however, have not been studied.

Oxidative stress can be induced in cell culture by either adding oxidative agents, by using medium without anti-oxidants [21-23], or by depriving cells of serum [24]. Some investigators have used mixed retinal cell cultures to assess the neuroprotective effects of drugs against various kinds of damage to RGCs [10,14]. However, it is difficult to exclude the latent mutual influence of other retinal cells on RGCs by this method [25]. On the other hand, purified cultured RGCs provide a simpler way to examine the effect of an agent on RGCs themselves, excluding confounding influences from other retinal cells.

In the present study, we investigated the effects of timolol, betaxolol, carteolol, and nipradilol on oxidative stress induced by excluding anti-oxidants from the neuronal culture medium on purified cultured rat RGCs. Rather unexpectedly, we found that some of the tested β-blockers showed protective effects against oxidative stress in RGCs at concentrations as low as 10 nM.

## Methods

### Materials

The animals used in this study were treated in accordance with the ARVO Statement for the Use of Animals in Ophthalmic and Vision Research. Poly-L-Lysine, bovine serum albumin (BSA), L-glutamine, and human recombinant brain-derived neurotrophic factor (BDNF) and rat recombinant ciliary neurotrophic factor (CNTF) were obtained from Sigma (St. Louis, MO). The papain dissociation system was from Worthington Biochemical (Lakewood, NJ); mouse anti-rat SIRP [CD172a] monoclonal antibody (MAB 1407P) and mouse anti-rat and mouse Thy1.1 monoclonal antibody (MAB1406) were obtained from Chemicon International (Temecula, CA) Vybrant® apoptosis assay kit and Live/Dead viability cytotoxicity kit (L-3224) were obtained from Molecular Probes (Eugene, OR). Timolol maleate was obtained from Merck & Co., Inc (Rahway, NJ), betaxolol was from Alcon Inc.(Fortworth, TX), carteolol was from Otsuka Pharmaceutical (Tokyo, Japan) and nipradilol was from Kowa Ltd. (Tokyo, Japan). CA-074 Me, a proinhibitor for intracellular cathepsin B, and E-64-d, an inhibitor for thiol proteases (cathepsin B/H/L, calpain), were purchased from Peptide Institute, Inc. (Osaka, Japan). Other reagents were obtained from Invitrogen (Carlsbad, CA) unless noted. B-27 supplement minus anti-oxidants (AO-) was purchased from Gibco (Grand Island, NY).

### Purified rat retinal ganglion cell culture

RGC cultures were obtained from the retinas of 6- to 8-day-old Wistar rats, following the two step immuno-panning procedure [26,27]. Briefly, retinas were dissociated into cell suspensions using the papain dissociation system. Separately, 50 ml flasks and 50 ml tubes were incubated with anti-rat macrophage antibody (1:50 dilution) and anti-rat and mouse Thy1.1 antibody (1:300 dilution) in phosphate buffered saline (PBS), respectively, at 4 °C overnight. Antibodies were removed and the cell suspension was incubated in the anti-macrophage antibody coated flask for 1 h. Suspensions containing cells that did not adhere to this flask were transferred to tubes coated with Thy1.1 antibody for 1 h. Cells adhering to the tube (RGCs) were resuspended in serum-free neurobasal medium (Gibco) supplemented with 2% B27 supplement, BDNF (40 ng/ml), CNTF (40 ng/ml) and forskolin (10 μM) and seeded onto 13 mm coverslips, placed within 24-well plates. The coverslips had been autoclaved, coated with 0.05 mg/ml of poly-L-lysine (Sigma) overnight, rinsed twice with Hanks' buffered saline solution (HBSS) and then coated for 2 h with 1 μg/ml of laminin (Gibco). RGCs were cultured for 3 days under normoxic conditions (20% O_2_, 5% CO_2_, 37 °C) before each experiment in serum-free B27 complete medium containing neurobasal medium (Gibco) with 1 mM L-glutamine (Sigma), B27 supplement (Gibco), 40 ng/ml BDNF, 40 ng/ml rat CNTF, 10 μM forskolin. In order to acquire fully-isolated RGCs, the density of RGCs in each well was seeded at approximately 500 cells/cm^2^ [26,27].

### Oxidative stress

After 3 days of cultivation, control coverslips were moved to freshly prepared neurobasal medium with B27 supplement (AO+), while coverslips for oxidative treatment were moved to neurobasal medium containing B27 but without anti-oxidants (AO-). Regular B27 supplement contains potent antioxidants (reduced glutathione, vitamin E, vitamin E acetate, catalase and superoxide dismutase) but B27 without antioxidants (AO-) does not contain these 5 compounds [28]. This AO- medium was used to induce RGC damage by oxidative stress [23].

### Detection of necrotic and apoptotic cells

RGCs were incubated under AO+ or AO- for 2 h and then Alexa Fluor 488-conjugated annexin V binding, combined with propidium iodide labeling, was performed [29,30]. Apoptotic RGCs were stained as annexin V positive and propidium iodide negative (annexin V+/propidium iodide-), and necrotic ones were positively stained with both annexin V and propidium iodide (annexin V+/propidium iodide+). Undamaged RGCs remained negative for both stains [29,30]. At the end of the above double staining, Hoechst 33342 was added to the culture medium at 8 μM. Cells were counted in at least 10 random fields of each well at 200X magnification using a fluorescence microscope (Nikon Eclipse TE300, Tokyo, Japan). The percentages of apoptotic and necrotic RGCs were quantified by determining the ratio of annexin V+/propidium iodide- cells, and annexin V+/propidium iodide+ cells, to Hoechst 33342-positive RGCs. Additionally, RGCs cultured in AO+ conditions with the addition of staurosporine (final concentration, 30 μM) were simultaneously assessed as a positive control for apoptosis and necrosis [31]. RGCs cultured in AO- conditions with the addition of CA-074 Me, a proinhibitor for intracellular cathepsin B, or E-64-d, an inhibitor for thiol protease (cathepsin B/H/L, calpain; final concentration, 50 μM) were also simultaneously assessed.

### Application of β-blockers

For evaluating the effects of timolol, betaxolol, carteolol and nipradilol on the oxidative stress-induced damage of RGCs, cells were incubated in AO- medium with or without β-blockers for 24 h after 72 h incubation in AO+ medium. We preliminarily examined the effect of each blocker at 10 nM and compared to the control AO- condition. As a result, timolol was only effective among 4 blockers at 10 nM. Finally, considering the limited samples of primary culture of RGCs, 3 concentrations, 1, 10, and 100 nM for timolol and 10, 100, and 1000 nM for other 3 β-blockers, were applied to RGC culture for further 24 h.

### Assay of RGC survival rate

Following 24 h in the presence of β-blockers, RGCs were processed for viability by labeling with calcein-AM (2 μM), a component of the Live/Dead Viability/Cytotoxicity kit as documented previously [26]. Ethidium homodimer-1 (2.5 μM) labeling was used to observe dead cells. In the present study, live RGCs were defined as having a calcein-stained cell body with neurites extending at least 2 cell diameters from the cellular body. RGC viability in AO- group with or without β-blockers was calculated from 2 wells, normalized to control AO+ group cultivated in parallel under the same conditions, and indicated as RGC survival rate. The average cell survival percentage of 8 experiments for each condition was expressed in the text and figures as the mean±standard deviation (SD).

### Statistical analyses

One way ANOVA analysis followed by the Tukey test and Dunnett test were used in this study. All data are indicated as mean±SD.

## Results

### Oxidative stress

**Figure 1:**
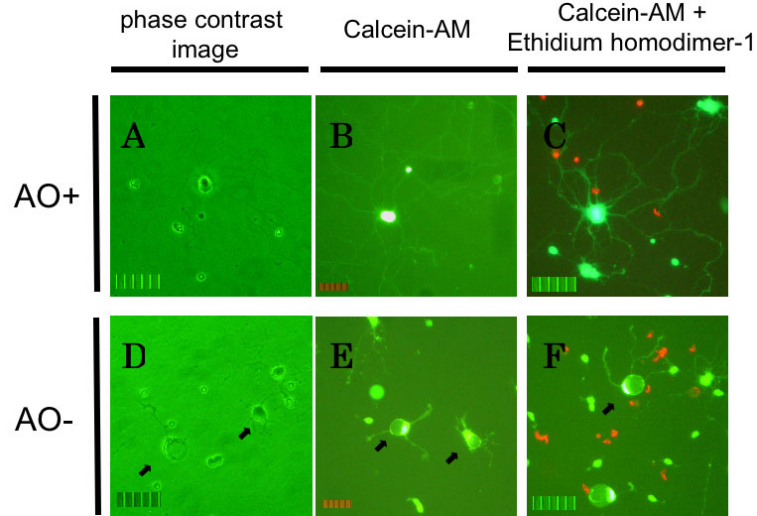
Rat retinal ganglion cell death by oxidative stress. Rat retinal ganglion cells (RGCs) after 72+24 h of culture under AO+ conditions (**A**, **B**, **C**) or AO- conditions (**D**, **E**, **F**). **A**, **D**: Phase contrast images of RGCs without labeling. **B**, **E**: Live cells with labeling of calcein-AM. **C**, **F**: Dead cells (red) with labeling of both calcein-AM and ethidium homodimer-1. The dendrites become shortened and cellular bodies had a deformed appearance (arrows). The scale bar represents 50 μm.

The effects of AO+ and AO- culture medium were assessed on RGCs in culture. The appearance of rat RGCs cultured under AO+ conditions is shown in Figure 1A-C. RGCs undergoing 3 days of AO+ culture and then transferred to AO- culture medium are shown in Figure 1D-F. Under oxidative stress for 24 h, the number of live RGCs decreased, the dendrites were shortened and their contours deformed.

### Detection of necrotic and apoptotic cells

**Figure 2:**
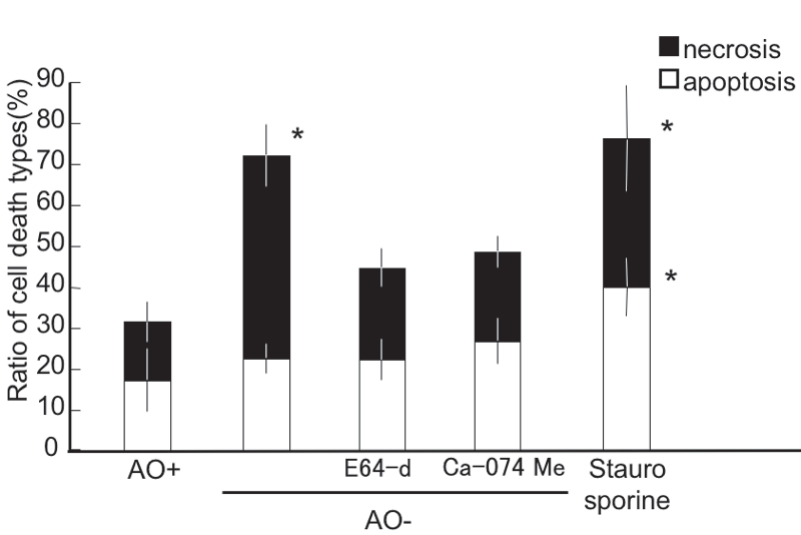
Detection of apoptotic and necrotic retinal ganglion cells under oxidative stress. Apoptotic retinal ganglion cells (RGCs) were significantly increased in the staurosporine-treated RGCs, but not in any other condition. Necrotic RGCs were significantly increased in the AO- and staurosporine conditions. E64-d and Ca-074 Me significantly reduced the necrotic cell percentage compared to the AO- conditions, but the values were not significantly different from the AO+ group. Each value represents mean±SD, (n=8). Asterisk indicates p<0.05, versus all the other groups (Tukey test).

The percentage of necrotic and apoptotic cells was evaluated for each condition (Figure 2). After 2 h incubation, the apoptotic RGCs percentages were 17.0±7.7% in the control AO+ group, 22.3±3.6% in the AO- group, 22.0±4.9% in the E-64-d group, 26.6±5.5% in the Ca-074 Me group, and 40.1±7.1% in the staurosporine-treated group. There was no significant difference between the AO+ group and the AO- group, while the apoptotic cell percentage in the staurosporine-treated group was significantly higher than all the other groups (p<0.05 by Tukey test). Both the cathepsin B proinhibitor (E-64-d) and thiol protease inhibitor (Ca-074 Me) added to AO- medium had no effect on apoptotic cell death. For necrotic RGCs, the percentages for the AO+, AO-, E-64-d, Ca-074 Me, and staurosporine-treated groups were 14.3±4.9%, 49.6±7.6%, 22.5±4.7%, 21.8±3.9%, and 36.3±13.1%, respectively. The percentage of necrotic cells in the AO- and staurosporine-treated groups were significantly higher than that of the AO+ control cells (p<0.05 by Tukey test). Necrotic RGCs percentages in the E-64-d and Ca-074 Me groups were significantly lower than that of the AO- group (p<0.05), but there was no significant difference from the AO+ group. These results indicated that AO- conditions increased necrotic cell death, but not apoptotic cell death.

### Effect of timolol

**Figure 3:**
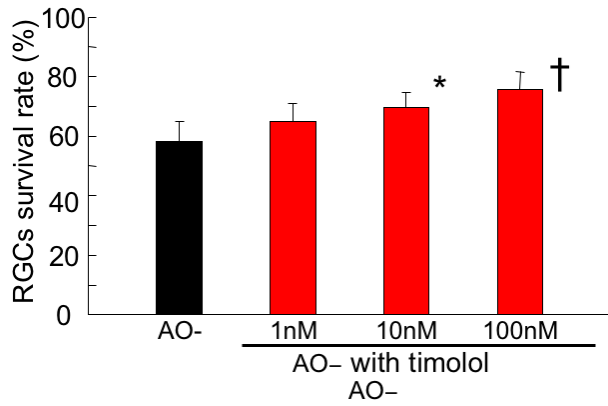
Effect of timolol on oxidative stress-induced retinal ganglion cell damage. Following 24 h under AO- condition in the presence of timolol, the survival rate of RGC was assessed and normalized to that of the control AO+ condition. At 10 and 100 nM, timolol significantly increased retinal ganglion cell viability. Data are expressed as mean±SD, n=8. Asterisk indicates p<0.05; cross symbol indicates p<0.01 by Dunnet test.

The effect of the non-selective β-blocker, timolol, on oxidative stress-induced RGC damage was investigated (Figure 3). In the control AO- group, RGC viability was 58.3 ±5.6%, while with 1 nM, 10 nM, and 100 nM timolol added it was 62.5±6.2%, 68.4 ±6.8%, and 75.2±6.5%, respectively (n=8). At 10 and 100 nM, timolol significantly increased RGC viability (Dunnet test, p<0.01-0.05).

### Effect of betaxolol

**Figure 4:**
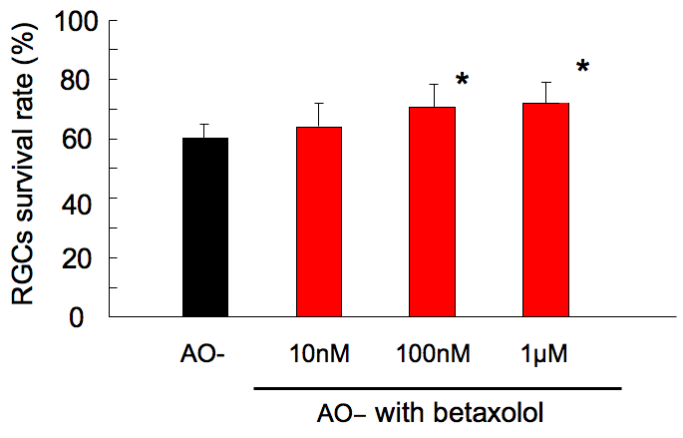
Effect of betaxolol on oxidative stress-induced retinal ganglion cell damage. Following 24 h under AO- condition in the presence of betaxolol, the survival rate of RGC was assessed and normalized to that of the control AO+ condition. At 100 nM and 1 μM, betaxolol significantly increased RGC viability. Data are expressed as mean±SD, n=8. Asterisk indicates p<0.05 by Dunnet test.

The effects of betaxolol on oxidative stress-induced RGC damage are summarized in Figure 4. Viability of RGCs without betaxolol and with 10 nM betaxolol was 59.8±4.8% and 63.2±8.8%, respectively. Betaxolol, 100 nM and 1 μM, significantly increased RGC viability to 69.2±8.6% and 71.0±7.2%, respectively. (Dunnet test, p<0.01-0.05).

### Effect of carteolol

**Figure 5:**
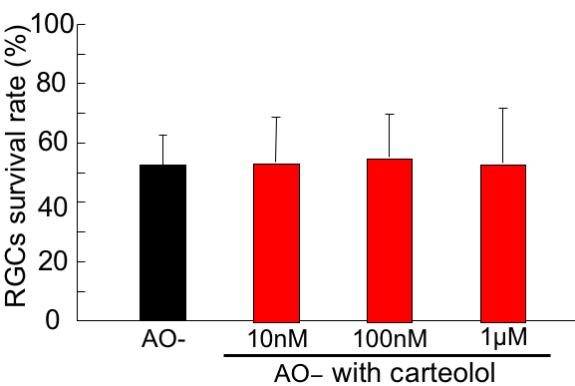
Effect of carteolol on oxidative stress-induced retinal ganglion cell damage. Following 24 h under AO- condition in the presence of carteolol, the survival rate of RGC was assessed and normalized to that of the control AO+ condition. Carteolol showed no significant effect on oxidative stress-induced damage of RGCs. Data are expressed as mean±SD, n=8.

Carteolol showed no significant effect on oxidative stress-induced damage of RGCs by Dunnet test (Figure 5). The viability of RGCs in AO- conditions, with the addition of 0, 10 nM, 100 nM, and 1 μM carteolol, was 52.5±9.9%, 53.2±15.3%, 55.0±14.4%, and 53.1±18.5%, respectively.

### Effect of nipradilol

**Figure 6:**
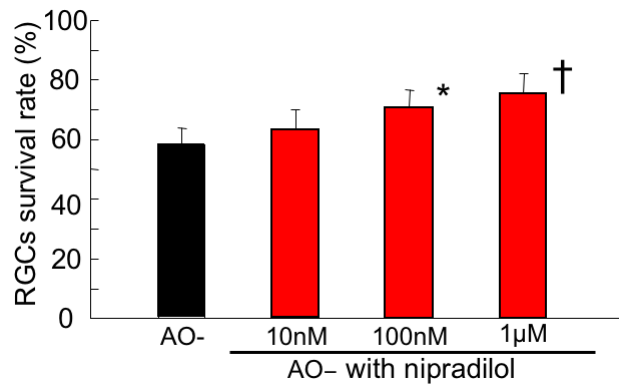
Effect of nipradilol on oxidative stress-induced retinal ganglion cell damage. Following 24 h under AO- condition in the presence of nipradilol, the survival rate of RGCs was assessed and normalized to that of the control AO+ condition. Nipradilol (100 nM and 1 μM) significantly increased RGC viability. Data are expressed as mean±SD, n=8. Asterisk (*) indicates p<0.05; christ symbol indicates p<0.01 by Dunnet test.

The effect of nipradilol on oxidative stress-induced RGC damage was investigated (Figure 6). At 100 nM and 1 μM, nipradilol significantly increased RGC viability to 69.3±7.2% and 73.7±7.1%, respectively, while it was 57.8±6.2% and 63.0±7.2% without nipradilol and with 10 nM nipradilol, respectively. (Dunnet test, p<0.01-0.05).

## Discussion

In the present study, B27 supplement without the anti-oxidants glutathione, vitamin E, vitamin E acetate, catalase, and superoxide dismutase, was used as an oxidative insult. The relative contributions of necrotic and apoptotic cell death depend on several factors, including the energy content of the cell and the severity of the insult [32]. Contrary to apoptosis, necrosis is not programmed cell death but occurs when cells are exposed to extreme stress [33]. For example, high concentrations of peroxynitrite induce necrosis in cortical neuron cultures, while at low concentrations it induced apoptosis [34]. Moreover, intracellular signaling pathways activated by various oxidative stresses are complicated, and there may be cross-talk between different pathways that, results in cell death. In the retinal ganglion cell line RGC-5, H_2_O_2_- or *tert*-butyl peroxide-induced cell death did not involve the classic apoptotic pathway, but caused glutathione depletion, which in turn generated a high concentration of glutamate, that induced DNA fragmentation as ROS did not scavenged [35]. These reports indicate that necrosis and apoptosis may be induced simultaneously and often are not easily distinguished. Detection of the type of cell death in our oxidative stress model showed that necrotic cell death was predominantly induced in AO- conditions. This was inhibited by the protease inhibitors of necrotic cell death, calpain or cathepsin. The current results suggest that depleting anti-oxidants might provide a stronger insult than other oxidative stress models and that B27 without anti-oxidant could be useful for investigating the mechanism of necrotic cell death with and without prior treatment with drugs. In this oxidative stress model, timolol showed a significant dose-dependent protective effect at a concentration on 10 nM and higher, while betaxolol and nipradilol also were found to be protective at 100 nM and higher.

Little is known about the molecular mechanisms required to induce necrotic cell death. Proteolysis is thought to play an important role in necrosis, and caspases, calpain, and cathepsins are mainly involved in necrotic cell death and neurodegeneration [33]. All these proteases can be activated directly or indirectly by an excessive increase of intracellular calcium. In fact, it was also reported that an increase of intracellular Ca^2+^ was associated with oxidative damage to cells [2,3]. Furthermore, oxidative stress may cause a Na^+^ influx concurrently, through L-type voltage-sensitive Ca^2+^ channels [36]. Thus, it is possible that the protective effect of some β-blockers may be explained based on the calcium induced proteolytic signals initiated by oxidative stress.

The neuroprotective effects of timolol and betaxolol on glutamate-, anoxia- or ischemia-induced insults have been reportedly attributed to their inhibition of Ca^2+^ and/or Na^+^ influx [10-12,14,37-40]. Nipradilol may also be able to reduce the influx of Ca^2+^ into the neuron through an α-1 adrenoceptor antagonistic effect [41]. However, according to previous studies, the IC50 for these effects was 1 μM or higher [12,41,42]. Since timolol was effective at 10 nM and betaxolol and nipradilol at 100 nM, the protective effects against oxidative stress-induced RGC damage encountered in this study are difficult to be attributed to Ca^2+^ and/or Na^+^ influx. β-blocking alone is also unlikely to be related since carteolol showed no effect.

Although the exact mechanism is unclear, the current study shows that betaxolol, timolol, and nipradilol could dose-dependently improve the survival rate of cultured purified RGCs under oxidative stress, at concentrations of 10 nM or 100 nM. Several previous studies showed that betaxolol, timolol, or nipradilol penetrates normal rabbits' ipsilateral retina at 100 nM when these agents were topically applied [12,13,20]. It must be noted however that direct extrapolation of results obtained in rabbits to humans is difficult. Recently, Grover et al reported that one of topical IOP-lowering drugs inhibiting carbonic anhydrase, dorzolamide, caused a significant decrease in retinal thickness in retinitis pigmentosa complicated with cystoid macular edema in humans [43]. Since the concentration of dorzolamide in plasma should not be high enough to cause any pharmacological effects attributable to inhibition of carbonic anhydrase [44], this effect may be attributable to dorzolamide locally penetrating the posterior fundus after instillation. Therefore, in addition to their IOP-lowering effects, some β-blockers directly alleviate oxidative stress-induced RGC damage in vivo.

In conclusion, oxidative stress induced RGC death were alleviated by some of beta-blockers. Retina and retinal neurons are susceptible to oxidative stress because these tissues consume high oxygen and exposed to light, and oxidative stress are related to GON [1,4-6]. Thus, β-blockers are expected to have neuroprotective effect in addition to IOP reduction. Although the neuroprotective mechanism of β-blockers for oxidative stress is still unknown, this additive effect may deserve future studies.
